# New cave-dwelling species of Tomoceridae from China, with a study on the pattern of mesothoracic bothriotricha in Tomocerinae (Collembola, Entomobryomorpha)

**DOI:** 10.3897/zookeys.574.7312

**Published:** 2016-03-28

**Authors:** Daoyuan Yu, Qibao Yan, Manqiang Liu

**Affiliations:** 1 Soil Ecology Lab, College of Resources and Environmental Sciences, Nanjing Agricultural University, Nanjing 210095, P. R. China; 2 Jiangsu Collaborative Innovation Center for Solid Organic Waste Resource Utilization, Nanjing 210014, P. R. China; 3 Department of Entomology, College of Plant Protection, Nanjing Agricultural University, Nanjing 210095, P. R. China

**Keywords:** *Tomocerus*, *Monodontocerus*, troglobitic, Tomocerinae

## Abstract

Two new troglobitic species of Tomoceridae are described from Guizhou and Guangxi provinces, China. *Tomocerus tiani* **sp. n.** resembles *Tomocerus kinoshitai* Yosii, 1954, *Tomocerus caecus* Yu & Deharveng, 2015 and *Tomocerus similis* Chen & Ma, 1997 but differs from them mainly in the body colour, the cephalic chaetotaxy and the number of manubrial pseudopores. *Monodontocerus cinereus* **sp. n.** is similar to *Monodontocerus mulunensis* Yu, Deharveng & Zhang, 2014 but is different from the latter in the body colour, the length of antennae, the number of ungual teeth and the chaetotaxy on Abd. III and Abd. IV. Special remarks are made on the mesothoracic bothriotricha in Tomocerinae.

## Introduction

Since the discovery of *Tritomurus scutellatus* Frauenfeld, 1854 in Slovenian caves, troglobitic Tomoceridae have frequently been reported in Asia, Europe and North America. Besides the troglobitic genera *Tritomurus* Frauenfeld, 1854 and *Lethemurus* Yosii, 1970, some other main groups of Tomoceridae have also been found with cave dwellers, and several genera, i.e. *Monodontocerus* Yosii, 1955, *Plutomurus* Yosii, 1956 and *Aphaenomurus* Yosii, 1956 have mainly or usually been found in caves. However, despite of several highly troglomorphic species, for example *Tritomurus falcifer* Cassagnau, 1958 and *Tritomurus veles* Lukić, Houssin and Deharveng, 2010 which are both eyeless and have very elongated antennae and claws, other cave tomocerids do not exhibit distinct troglomorphic characters: most of them have short to moderate antennae, normal or slightly elongated claws and a full set of 6+6 eyes for Tomocerinae, only the sizes of eyes are usually smaller than those of the edaphic species, and the tenent hair is often pointed as in other cave Collembola.

Four cave tomocerids have previously been reported in China, including *Tomocerus caecus* Yu & Deharveng, 2015, *Monodontocerus absens* Yu, Deharveng & Zhang, 2014 and *Monodontocerus mulunensis* Yu, Deharveng & Zhang, 2014 from Guangxi Province, and *Monodontocerus trigrandis* Yu, Deharveng & Zhang, 2014 from Hunan Province. The present paper reports two new species of Tomoceridae discovered in caves in the south-west karst regions of China. Both of the new species have small eyes and pointed tenent hair, but neither of them is highly troglomorphic. *Tomocerus tiani* sp. n. bears some unusual characters, including the single mesothoracic bothriotrichum, which lead to a comprehensive examination of the different genera of Tomocerinae.

## Materials and methods

Specimens were collected with aspirators and preserved in 99% ethanol. For detailed morphological study, specimens were cleared in Nesbitt’s fluid and mounted in Marc André II solution. For some specimens the furca, the ventral tube and the legs were cut off from the trunk and mounted separately for detailed observation. The slide-mounted specimens were studied using a Nikon Ni microscope. Photos were taken using Nikon DS-Fi1 cameras mounted respectively on Nikon SMZ1000 stereomicroscope and Nikon Ni microscope.

Fjellberg (2007) is followed for maxillary lamellae numbering, Yu et al. (2014) for the pattern of cephalic dorsal chaetotaxy and Christiansen (1964) for body macrochaetotaxy. The description of the body chaetotaxy refers to one side only since in most case it is symmetric. The exact morphology of each chaeta was unclear due to shedding. The dental spines formula follows that of Folsom (1913), in which the dental spines are arranged from basal to distal, with a slash indicating the separation between basal and medial subsegments and the Roman numerals referring to spines that are noticeably larger. If not mentioned specially, all descriptions are based on fully developed individuals.

### Abbreviations

Ant.
 antennal segment 


PAO
 postantennal organ 


Th.
 thoracic segment 


Abd.
 abdominal segment 


**Institutional acronyms**

NJAU
 Nanjing Agricultural University, Nanjing, China 


## Results

### Tomocerus tiani

Taxon classificationAnimaliaCollembolaTomoceridae

Yu sp. n.

http://zoobank.org/722C6CC5-F644-477E-91D4-5BFC6759A17B

Figs 1A, B , 2 , 3

#### Type-locality.

China, Guizhou Province: Zunyi, Suiyang County, Wenquan Town, Guihua Village, Hejiao Cave, inside cave, 7 November 2008, Mingyi Tian leg.

#### Type-specimens.

Holotype male (labelled 15cave15-1) and paratype juvenile (labelled 15cave15-2) on slide. Deposited in NJAU.

#### Diagnosis.

Species similar to *Tomocerus kinoshitai* Yosii, 1954, with short antennae, multi-furcated dental spines and apically curved mucro. Body length approximately 3.0 mm, with purplish grey pigment all over; antennae approximately half as long as body; eyes small; terminal hair of maxillary outer lobe with a small basal denticle; Th. II with only one bothriotrichum; tenent hairs pointed; unguis with two teeth, baso-internal ridges at approximately 1/2 distance from base; manubrium with 12–17 pseudopores on each side; dental spines formula as 4/2, II; dens dorsally with only a few feather-like chaetae; mucro without intermediate tooth. Cave-dwelling species.

#### Description.

**Figure 1.**
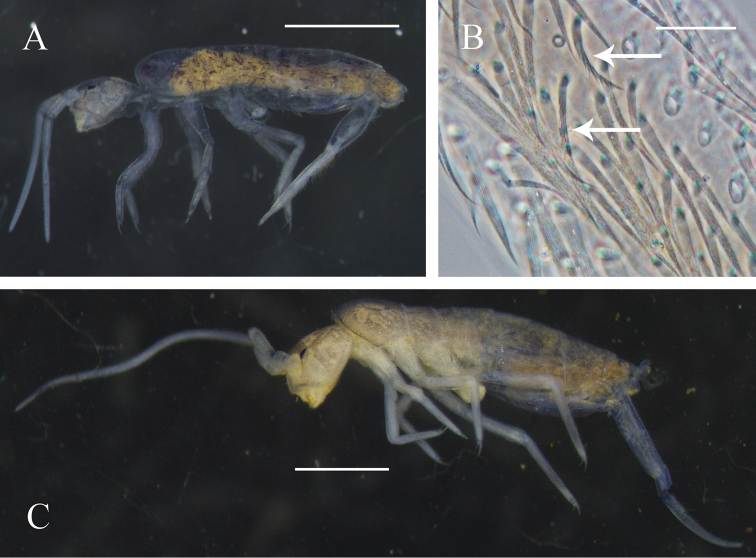
*Tomocerus tiani* sp. n. and *Monodontocerus cinereus* sp. n. **A** and **B**, *Tomocerus tiani* sp. n. **A** appearance in alcohol (lateral view) **B** dorsal chaetae on dens (dorsal view, arrows pointing to feather-like chaetae) **C** *Monodontocerus cinereus* sp. n. **C** appearance in alcohol (lateral view). Scale bars: 1000 μm (**A, C**), 50 μm (**B**).

Body length 2.9 mm. Body with uniform purplish grey pigment and unpigmented patches, appendages paler. Eye patches black (Fig. 1A). Types of scales and chaetae typical for Tomocerinae.

**Figure 2.**
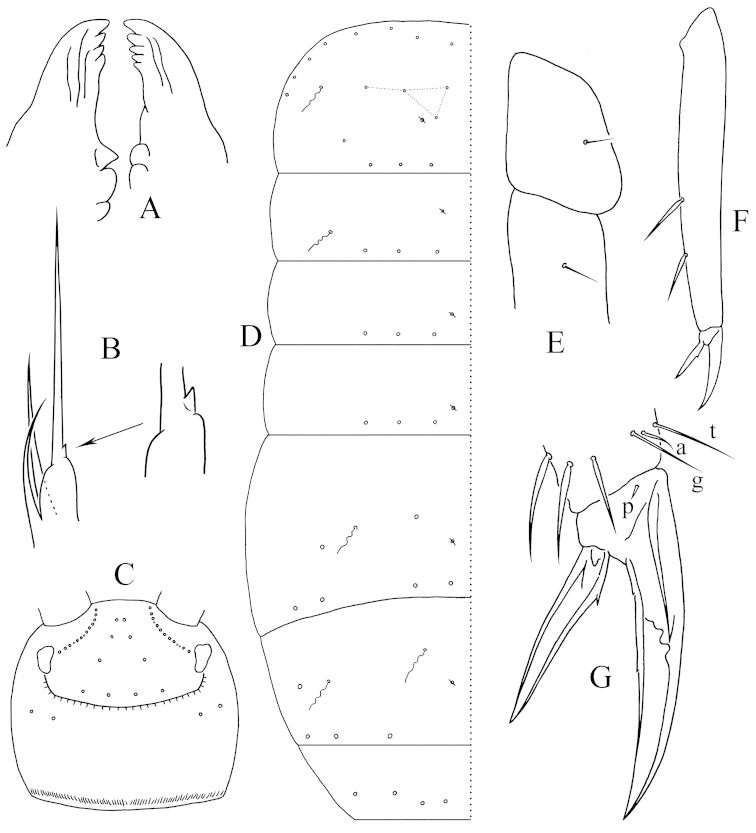
*Tomocerus tiani* sp. n. **A** mandibular heads (dorsal view) **B** palp of left maxillary outer lobe (dorsal view); **C** cephalic dorsal chaetotaxy (dorsal view, circle: socket of chaeta, same as below) **D** dorsal chaetotaxy of Th. II–Abd. V (dorsal view, circle with a slash: pseudopore, wavy line: bothriotricha, same as below) **E** trochantero-femoral organ (inner view) **F** hind tibiotarsus (lateral view, showing spine-like inner chaetae) **G** hind claw (lateral view, t: tenent hair, a: accessory chaeta, g: guard chaeta, p: pretarsal chaeta, same as below).

Antennae approximately half length of body. Length ratio of antenna segments as I:II:III+IV = 1.0:1.9–2.0:9.6–9.7. Only dorsal side of Ant. I and Ant. II scaled, Ant. III+IV unscaled. PAO not seen. Eyes 6+6, relatively small. Labral formula as 4/5, 5, 4. Distal edge of labrum with four curved spine-like papillae. Mandibular heads asymmetrical, the left one with four teeth and the right one with five, left molar plate distally with a tapered tooth (Fig. 2A). Maxillary lamella five without beard-like appendage. Maxillary outer lobe with trifurcate palp, one basal chaeta and four sublobal hairs; terminal hair with a small basal denticle (Fig. 2B). Both dorsal and ventral sides of head scaled. Cephalic dorsal macrochaetotaxy: anterior area: 2, 2; interocular area: 2, 4, central uneven macrochaeta absent; postocular area: 2+2; posterior area: 0. Posterior margin of head with ca. 70 small chaetae (Fig. 2C). Mentum with five chaetae, submentum with numerous chaetae.

Pattern of body chaetotaxy as in Fig. 2D. Number of bothriotricha as 1, 1/ 0, 0, 1, 2, 0, 0 on Th. II–Abd. VI. Macrochaetae densely arranged along anterior margin of Th. II (not shown in figure). Th. II with a row of macrochaetae behind anterior margin. Number of macrochaetae or large mesochaetae in the posterior row as 3, 3/ 3, 3, 4, 3, 4 (3 dorsal+1 lateral) from Th. II to Abd. V. Th. II with four central and one lateral macrochaetae, postero-central chaeta near pseudopore; Abd. III with two anterior macrochaetae; Abd. IV with one lateral macrochaeta; Abd. VI with numerous chaetae of moderate size. Most mesochaetae laterally and posteriorly on terga. Pseudopores near the axis of terga, number of them as 1, 1/ 1, 1, 1, 1, 0, 0 from Th. II to Abd. VI.

Trochantero-femoral organ with 1, 1 small slender chaetae (Fig. 2E). Front, middle and hind tibiotarsus ventrally with 0, 0, 2 pointed spine-like chaetae (Fig. 2F). Each tibiotarsus with a distal whorl of 11 chaetae, ventral six as ordinary chaetae, dorsal five modified: tenent hair thin and pointed, approximately 0.33 times as long as inner edge of unguis; two accessory chaetae small, longer than pretarsal chaetae; two guard chaetae of same morphology and size as tenent hair. Unguis slender, with baso-internal ridges at approximately 1/2 distance from base; lateral teeth pointed, of moderate size. Inner edge of unguis with one basal and one central minute teeth. Unguiculus rather slender, approximately 0.5–0.72 times as long as unguis, its inner edge with one corner tooth. Pretarsus chaetae 1+1 (Fig. 2G).

**Figure 3.**
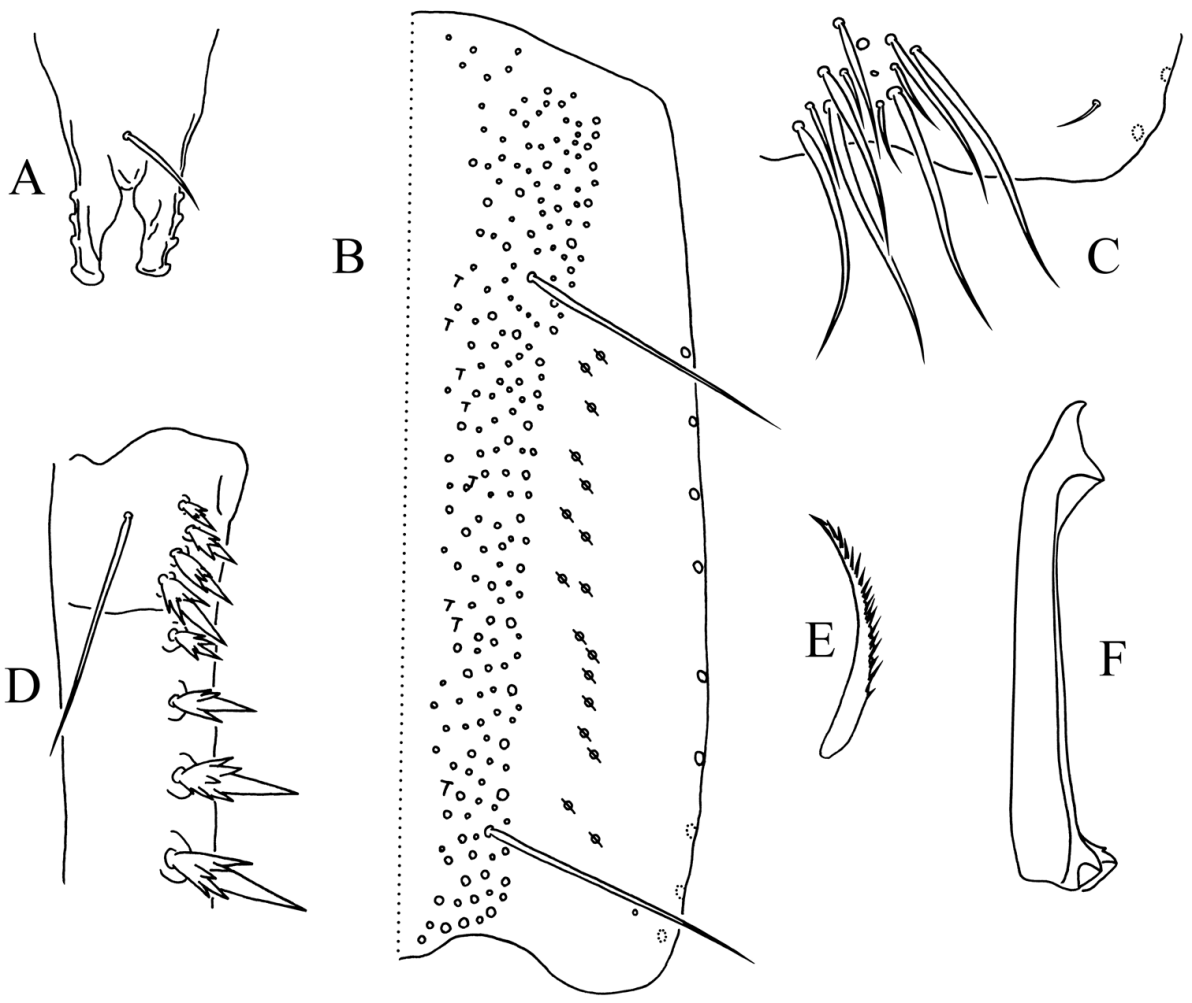
*Tomocerus tiani* sp. n. **A** tenaculum (anterior view) **B** right side of manubrium (dorsal view, showing prominent dorsal chaetae, T-shaped mark: socket of scale, same as below) **C** disto-external corner of manubrium (dorsal view) **D** basal and middle subsegments of dens (dorsal view, showing dental spines and prominent dorso-basal chaeta) **E** feather-like dental chaeta (lateral view) **F** mucro (lateral view).

Anterior face of ventral tube with scales, posterior face and lateral flaps unscaled, anterior face with ca. 25 chaetae on each side, posterior face with ca. 30 chaetae, each lateral flap with ca. 15 chaetae. Rami of tenaculum with 4+4 teeth, anterior face with one chaeta and without scale (Fig. 3A). Length ratio of furca segments as manubrium : dens : mucro = 2.5 : 3.6–3.7 : 1.0. Manubrium ventrally scaled, without chaetae, laterally with large round scales and 7–9 strong chaetae; dorsal chaetal stripe with ca. 200 chaetae of different sizes, including 2+2 pointed prominent chaetae; inner side of chaetal stripe with several scales; pseudopores 12–17 on each side (Fig. 3B); external corner chaeta as a microchaeta (Fig. 3C). Dens basally with a pointed prominent dorsal chaeta, without large modified inner scale or strong outer chaetae. Dental spines formula as 4/2, II; spines with moderate to large sized denticles around basal half (Fig. 3D). Dens dorsally with ordinary chaetae, swollen spine-like ciliated chaetae and a few feather-like chaetae (Figs 1B, 3E), ventrally with scales and several apical chaetae. Mucro elongated, distally curved and multi-setaceous; both basal teeth with proximal lamellae, outer tooth with a toothlet; apical and subapical tooth subequal; two dorsal lamellae running from subapical tooth, outer lamella ending in inner basal tooth, inner lamella ending at base of mucro; without intermediate teeth (Fig. 3F).

#### Etymology.

Named after the collector Prof. Mingyi Tian.

#### Remarks.

*Tomocerus tiani* sp. n. is similar to *Tomocerus caecus* Yu & Deharveng, 2015, *Tomocerus kinoshitai* (materials from Changbai Mountain, China) and *Tomocerus similis* Chen & Ma, 1997 (type materials) in the length of antennae, the general pattern of chaetotaxy on Th. II, the number and position of spine-like tibiotarsal inner chaetae, the size of external corner chaeta on the manubrium, the type and general arrangement of dental spines and the shape of mucro, but it is clearly different from the first species in having eyes and pigment, and is different from the other two species mainly in the body colour, the cephalic chaetotaxy, the sharply pointed tibiotarsal strong inner chaetae and tenent hair, and the more slender unguiculus; besides, with similar body size, *Tomocerus tiani* sp. n. has more manubrial pseudopores than the three known species. The small basal denticle of the terminal hair of maxillary outer lobe is so far unique to *Tomocerus tiani* sp. n. and is useful for diagnosis if dissected and exposed carefully. The baso-internal ridges of unguis are located at approximately 1/2 distance from the base in *Tomocerus tiani* sp. n., whereas in most other species the distance between the ridges and the base is only 1/3 or less. The dorsal dental chaetae in *Tomocerus tiani* sp. n. is also characteristic, that the dense stripes of feather-like chaetae in most other tomocerids are almost replaced by ordinary chaetae and swollen serrated chaetae, leaving only a few feather-like ones. Similar condition was also reported in *Tomocerus kinoshitai* and *Tomocerus caecus* that some spine-like chaetae are present on dens (Yosii 1967, Yu and Deharveng 2015).

The juvenile specimen is almost identical to the adult in most characters, including the macrochaetotaxy, the number of teeth on claws and the dental spines formula. However, some characters on manubrium are distinctly different between juvenile and adult. In the juvenile specimen, there are ca. 80 dorsal chaetae on each side of manubrium, the number of pseudopores is only 4–5 on each side, and the external corner chaeta is as large as a mesochaeta in the dorsal chaetal stripe. These differences provide interesting information in the postembryonic development of manubrium in Tomocerinae, and indicate these characters are not suitable for the identification of immature specimens at different instars.

### Monodontocerus cinereus

Taxon classificationAnimaliaCollembolaTomoceridae

Yu sp. n.

http://zoobank.org/7C9C6A04-387B-4558-ADCC-B9C0CF7B8F2F

Figs 1C , 4 , 5

#### Type-locality.

China, Guangxi Province: Hechi, Duan County, Chengjiang Township, Ganwan Village, Nongsi Cave, 23°56'24"N, 108°10'12"E, alt. ca. 270 m, inside cave, 25 July 2015, Jujian Chen, Xinhui Wang and Mingruo Tang leg.

#### Type-specimens.

Holotype male (labelled 15cave6-1) and two paratypes female (labelled 15cave6-2 and -3) on slides, one paratype (labelled 15cave6) in alcohol. Deposited in NJAU.

#### Diagnosis.

Typical *Monodontocerus* species with multi-furcated dental spines and single mucronal basal tooth. Body length approximately 4.0 mm, with light grey pigment all over; antennae slightly shorter than body; eyes small; chaetotaxy typical for the genus; tenent hair pointed; unguis with 2–4 teeth; manubrium with 28–35 pseudopores on each side; dental spines formula as 4, II/6, I, 3, I or 5, I/6, I, 3, I; mucro with 3–4 intermediate teeth. Cave-dwelling species.

#### Description.

Body length 3.9–4.1 mm. Body colour uniformly light grey with unpigmented patches, appendages paler. Eye patches black. (Fig. 1C). Scales and chaetae of Tomocerinae type.

**Figure 4.**
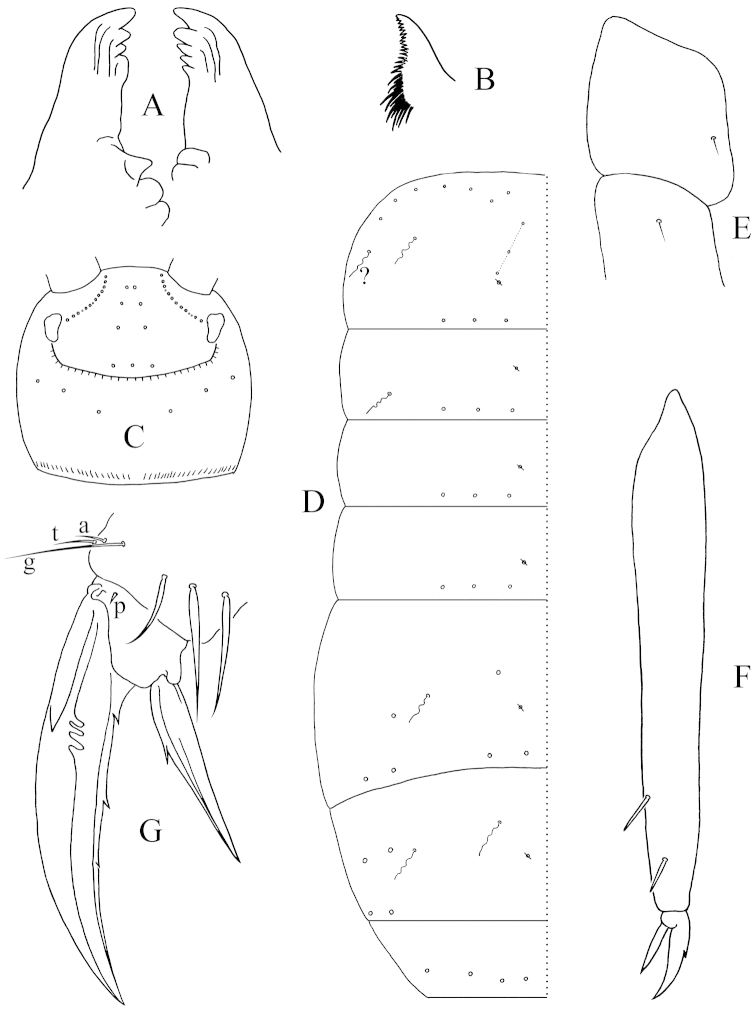
*Monodontocerus cinereus* sp. n. **A** mandibular heads (dorsal view) **B** maxillary lamella 5 (dorsal view) **C** cephalic dorsal chaetotaxy (dorsal view) **D** dorsal chaetotaxy of Th. II–Abd. V (dorsal view) **E** trochantero-femoral organ (inner view) **F** hind tibiotarsus (lateral view, showing spine-like inner chaetae) **G** middle claw (lateral view).

Antennae approximately 0.7–0.9 times as long as body. Length ratio of antenna segments as I:II:III:IV = 1.0:1.7–2.0:10.5–11.0:1.8. Only dorsal side of Ant. I and Ant. II scaled, Ant. III and Ant. IV unscaled. PAO not seen. Eyes 6+6, relatively small. Mouthparts typical for Tomocerinae. Labral formula as 4/5, 5, 4. Distal edge of labrum with four curved spine-like papillae. Mandibular heads asymmetrical, the left one with four teeth and the right one with five, left molar plate distally with a tapered tooth (Fig. 4A). Maxillary lamella five without beard-like appendage, basal teeth elongated (Fig. 4B). Maxillary outer lobe with trifurcate palp, one basal chaeta and four sublobal hairs. Both dorsal and ventral sides of head scaled. Cephalic dorsal macrochaetotaxy: anterior area: 2, 2; interocular area: 2, 3, central uneven macrochaeta present; postocular area: 2+2; posterior area: 2. Posterior margin of head with 40–50 small chaetae (Fig. 4C). Mentum with five chaetae, submentum with numerous chaetae.

Pattern of body chaetotaxy as in Fig. 4D. Number of bothriotricha as 2 (1?), 1/ 0, 0, 1, 2, 0, 0 on Th. II–Abd. VI. Macrochaetae densely arranged along anterior margin of Th. II (not shown in figure). Th. II with a row of macrochaetae behind anterior margin. Number of macrochaetae or large mesochaetae in the posterior row as 3, 3/ 3, 3, 4, 2, 4 (3 dorsal+1 lateral) from Th. II to Abd. V. Th. II with three central macrochaetae arranged approximately in a line, postero-central chaeta near pseudopore; Abd. III with two anterior macrochaetae; Abd. IV with two lateral macrochaetae; Abd. VI with numerous chaetae of moderate size. Most mesochaetae laterally and posteriorly on terga. Pseudopores near the axis of terga, number of them as 1, 1/ 1, 1, 1, 1, 0, 0 from Th. II to Abd. VI.

Trochantero-femoral organ with 1, 1 small slender chaetae (Fig. 4E). Front, middle and hind tibiotarsus ventro-distally with 0, 0, 2 blunt spine-like chaetae (Fig. 4F). Each tibiotarsus with a distal whorl of 11 chaetae, ventral six as ordinary chaetae, dorsal five modified: tenent hair very small and pointed; two accessory chaetae subequal to tenent hair, longer than pretarsal chaetae; two guard chaetae thin and pointed, approximately three times as long as tenent hair. Unguis slender, with baso-internal ridges approximately 1/3 distance from base; lateral teeth pointed, of moderate size. Inner edge of unguis with one basal and 1–3 more distal teeth. Unguiculus slender, approximately 0.6–0.7 times as long as unguis, its inner edge with 1–2 corner tooth. Pretarsus chaetae 1+1 (Fig. 4G).

**Figure 5.**
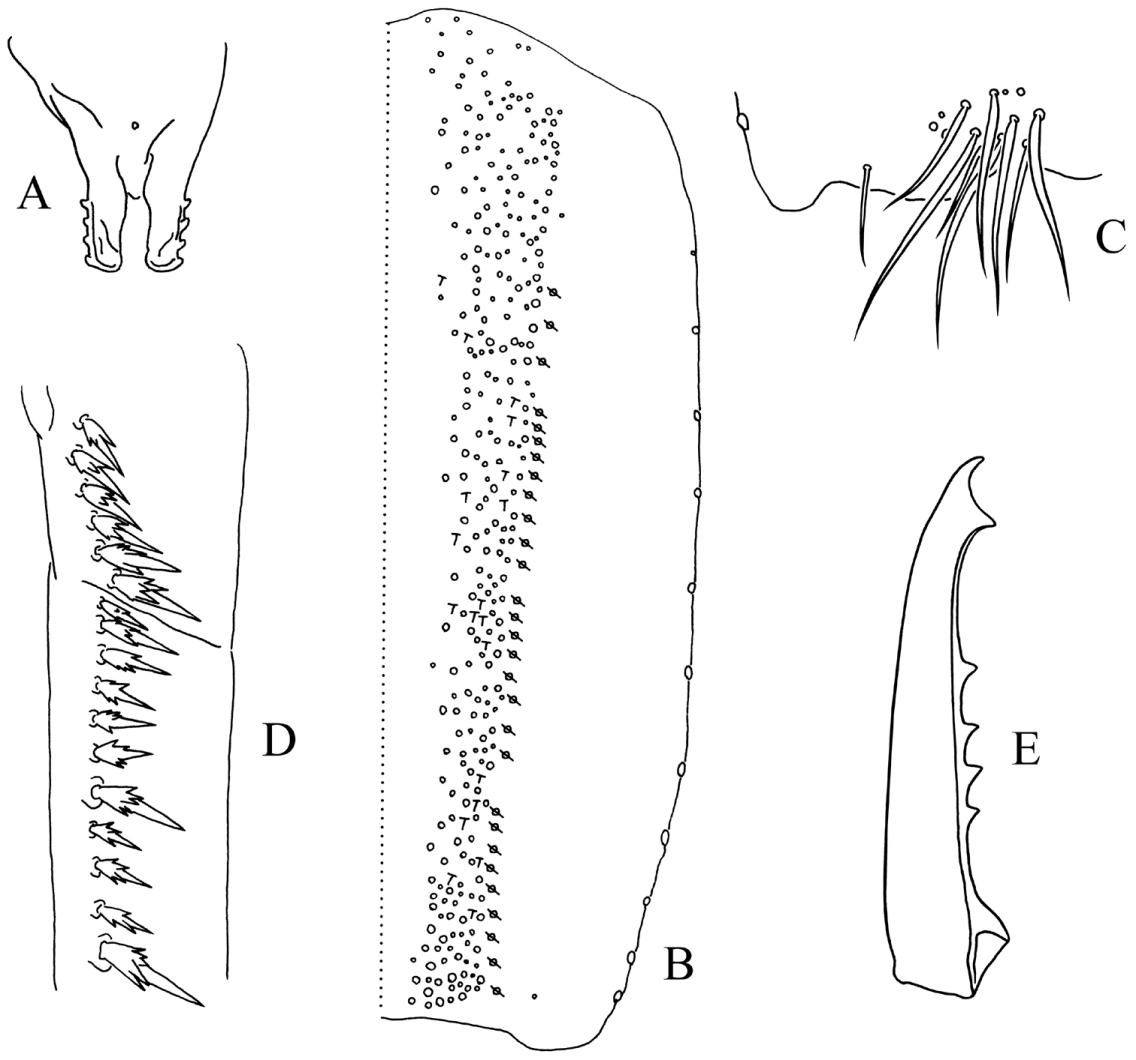
*Monodontocerus cinereus* sp. n. **A** tenaculum (anterior view) **B** right side of manubrium (dorsal view) **C** disto-external corner of manubrium (dorsal view) **D** basal and middle subsegments of dens (showing dental spines) **E** mucro (lateral view).

Ventral tube with scales on both anterior and posterior faces, lateral flaps unscaled, anterior face with ca. 70 chaetae on each side, posterior face with ca. 160 chaetae, each lateral flap with ca. 100 chaetae. Rami of tenaculum with 4+4 teeth, anterior face with one chaeta and without scale (Fig. 5A). Length ratio of furca segments as manubrium : dens : mucro=3.7–3.8 : 5.1–5.3 : 1.0. Manubrium ventrally scaled, without chaetae, laterally with large round scales and 9–10 strong chaetae; dorsal chaetal stripe with ca. 280 chaetae of different sizes, without distinct prominent chaetae; dorsal scales mixed with chaetae in chaetal stripe; pseudopores 28–35 on each side (Fig. 5B); external corner chaeta as a small mesochaeta in chaetal stripe (Fig. 5C). Dens basally without large modified inner scale or strong outer chaetae. Dental spines formula as 4, II/6, I, 3, I or 5, I/6, I, 3, I, proximal spines enlarged gradually; each spine consisting of a strong trunk surrounded by several denticles near base (Fig. 5D). Dens dorsally with ordinary chaetae and feather-like chaetae, ventrally with scales. Mucro elongated and multi-setaceous; single basal tooth with proximal lamella; apical and subapical tooth subequal; two dorsal lamellae running from subapical tooth, outer lamella ending at basal tooth, inner lamella ending at base of mucro; outer lamella with 3–4 moderate sized intermediate teeth (Fig. 5E).

#### Etymology.

Named for its light grey body colour, from the Latin *cinereus*, meaning ash-coloured.

#### Remarks.

Within the genus, *Monodontocerus cinereus* sp. n. is more similar to *Monodontocerus mulunensis* Yu, Deharveng & Zhang, 2014 in the cephalic chaetotaxy, the pointed tenent hair and the number of mucronal intermediate teeth, but can be distinguished from the latter by having longer antennae, fewer teeth on unguis and more macrochaetae on Abd. III and Abd. IV. In alcohol the new species can be identified from other known species of *Monodontocerus* by the grey body colour.

## Discussion

*Tomocerus tiani* sp. n. has only one bothriotrichum on Th. II, while there are two in most other *Tomocerus* species observed previously. The pattern of bothriotricha is significant for the taxonomy and phylogeny of Collembola (Szeptycki 1979, Soto-Adames et al. 2008). However, the exact pattern of mesothoracic bothriotricha in Tomocerinae has not been resolved though the number of them was commonly reported as either two (Yosii 1956, 1967, Fjellberg 2007) or one (Christiansen 1964, Chen and Ma 1997).

There are two main obstacles to determining the pattern of mesothoracic bothriotricha in Tomocerinae. Firstly, these long, thin and ciliated chaetae, as well as the macrochaetae, are easily lost during specimen collection and slide preparation, leaving only the sockets. The sockets of bothriotricha are usually characteristic for their round shape and small size, but sometimes can still be confused with the sockets of macro- or mesochaetae. Secondly, in case of two bothriotricha present they are arranged transversely, and the outer one is usually near the lateral margin of the tergum, thus could possibly be omitted when the margin is wrinkled or damaged. To avoid those disadvantages, we examined the specimens with almost intact coating of chaetae, most of which are pre-molting specimens with new chaetae under the old cuticle.

**Figure 6.**
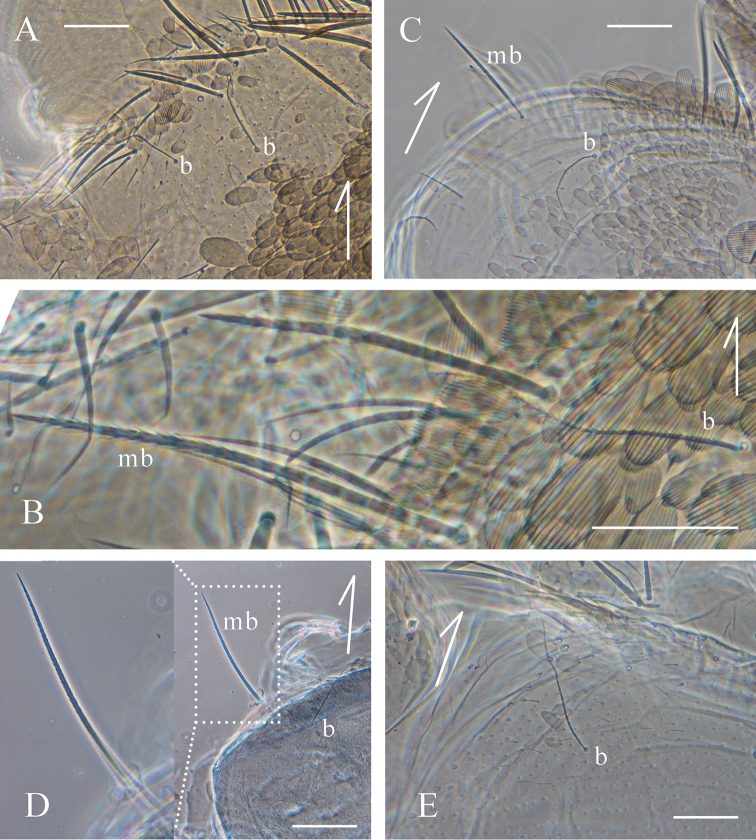
Dorsal view of the left antero-lateral corner of mesonotum (arrows directing anterior). **A** *Tomocerus* sp. (*ocreatus* complex) from China (b: bothriotricha, same as below) **B** *Tomocerus similis* from China (mb: macrochaeta at the location of outer bothriotricha, same as below) **C** *Tomocerina annamitica* from Vietnam **D** *Tomocerus laxalamella* from China (with bothriotricha-like macrochaeta two times magnified) **E** *Pogonognathellus* sp. from France. Scale bars: 50 μm (**A, B, C, E**), 100 μm (**D**).

In the observed species, there are three main patterns of mesothoracic bothriotricha, which appear to be relevant to the generic division except for several species of *Tomocerus* and *Tomocerina*. Pattern A: all species of *Tomocerus ocreatus* complex (Zhang et al. 2014), *Tomocerus nigrus* Sun, Liang & Huang, 2006 and *Tomocerus jilinensis* Ma, 2011 have two mesothoracic bothriotricha, and the socket of the inner one is usually larger, thus is more similar to that of a macrochaeta (Fig. 6A). Pattern B: *Tomocerus kinoshitai*, *Tomocerus similis*, *Tomocerina annamitica* Yu, Man & Deharveng, 2016, *Tomocerus laxalamella* Lee, 1975 (materials from Changbai Mountain, China), *Tomocerina varia* Folsom, 1899 (materials from Changbai Mountain, China) and *Tomocerina tianshanensis* Ma, Chen & Christiansen, 2003 have only one bothriotrichum at approximately the place of the inner bothriotrichum in other *Tomocerus*, and a macrochaeta is present instead of the outer bothriotrichum (Fig. 6B, C, D). In the first two species this macrochaeta is longer and apically more tapered than the adjacent macrochaetae (Fig. 6B); in *Tomocerina annamitica* this macrochaeta is slender (Fig. 6C), and its socket is very similar to that of a bothriotrichum; in the last three species the macrochaeta is large, rather elongated, pointed, and is more ciliated than other ordinary chaetae, forming a bothriotricha-like macrochaeta (Fig. 6D). Pattern C: in *Pogonognathellus heterochros* Wang, Yu & Zhang, 2013, *Pogonognathellus mai* Wang, Yu & Zhang, 2013 and *Pogonognathellus* sp. from France, there is only one bothriotrichum, and no distinctly special macro- or mesochaetae is present nearby (Fig. 6E).

This study has revealed several distinct patterns of mesothoracic bothriotricha in Tomocerinae, and has proved their taxonomic importance. However, since our study covered mostly Asian edaphic species, we have probably not exhausted the variability of this character among Tomocerinae. The exact pattern in *Monodontocerus* has not been successfully determined because of the lack of specimen with intact bothriotricha, though the sockets indicate pattern A more possible. For *Pogonognathellus* more examination is required since several species were described having two mesothoracic bothriotricha (Fjellberg 2007, Park et al. 2011). On the other hand, given the thoracic bothriotricha do not exist in the primary chaetotaxy (Szeptycki 1972, Yu et al. 2015), they could either be secondary elements or be transformed from certain primary chaetae. Tracing the postembryonic development of chaetotaxy will help to evaluate the true significance of thoracic bothriotricha for taxonomy, while study on the homology of chaetae will be a next step to review the current taxonomic system of Tomocerinae.

## Supplementary Material

XML Treatment for Tomocerus tiani

XML Treatment for Monodontocerus cinereus
